# Genetic Studies of Metabolic Syndrome in Arab Populations: A Systematic Review and Meta-Analysis

**DOI:** 10.3389/fgene.2021.733746

**Published:** 2021-11-18

**Authors:** Zahrah Al-Homedi, Nariman Afify, Mashal Memon, Habiba Alsafar, Guan Tay, Herbert F. Jelinek, Mira Mousa, Nadia Abu-Samra, Wael Osman

**Affiliations:** ^1^ College of Medicine and Health Sciences, Khalifa University, Abu Dhabi, United Arab Emirates; ^2^ Center for Biotechnology, Khalifa University, Abu Dhabi, United Arab Emirates; ^3^ Department of Genetics and Molecular Biology, College of Medicine and Health Sciences, Abu Dhabi, United Arab Emirates; ^4^ Department of Biomedical Engineering, College of Engineering, Khalifa University, Abu Dhabi, United Arab Emirates; ^5^ Department of Psychiatry, UWA Medical School, The University of Western Australia, Nedlands, WA, Australia; ^6^ School of Medical and Health Sciences, Edith Cowan University, Joondalup, WA, Australia; ^7^ Health Innovation Engineering Center, Khalifa University, Abu Dhabi, United Arab Emirates; ^8^ Nuffield Department of Women's and Reproduction Health, Oxford University, Oxford, United Kingdom; ^9^ Department of Biology, College of Arts and Sciences, Khalifa University, Abu Dhabi, United Arab Emirates

**Keywords:** metabolic syndrome, arab populations, genetic studies, systematic review, meta-analysis running title

## Abstract

**Background:** The metabolic syndrome (MetS) is prevalent in Arabian populations. Several small-scale studies have been performed to investigate the genetic basis of MetS. This systematic review and meta-analysis aimed to examine whether candidate gene polymorphisms are associated with MetS susceptibility among ethnic groups of the Arabian world and to suggest possible directions for future research regarding genetic markers and MetS.

**Methods:** A search was conducted for peer-reviewed articles that examined the genetic association of MetS in Arabian populations in the following databases: Medline, Embase, Scopus, Direct Science, Web of Science, ProQuest, and Google Scholar until March 31, 2021. Articles were eligible if they were case-control studies, which investigated MetS as a dichotomous outcome (MetS vs no MetS). To assess the quality of the studies, the Q-Genie tool (Quality of Genetic Association Studies) was used. A non-central chi2 (random-effect) distribution was used to determine the heterogeneity (H) of Q and *I* (Galassi et al., The American journal of medicine, 2006, 119, 812–819) statistics.

**Results:** Our search strategy identified 36 studies that met our inclusion criteria. In most cases, studies were excluded due to a lack of statistical information such as odds ratios, confidence intervals, and *p*-values. According to the Q-Genie tool, 12 studies scored poorly (a score of≤35), 13 studies scored moderately ( >35 and≤45), and 12 studies had good quality ( >45 or higher). The most frequently studied genes were FTO and VDR (both included in four studies). Three SNPs indicated increased risk for MetS after calculating the pooled odds ratios: FTO-rs9939609 (odds ratio 1.49, 95% CI: 0.96–2.32); LEP-rs7799039 (odds ratio 1.85, 95% CI: 1.37–2.5); and SERPINA12-rs2236242 (odds ratio 1.65, 95% CI: 1.21–2.24). Meta-analysis studies showed no significant heterogeneity.

**Conclusion:** There were many sources of heterogeneity in the study settings. Most of the studies had low to moderate quality because of sample size and power issues, not considering all potential sources of bias, and not providing details about genotyping methods and results. As most studies were small-scale, aimed to replicate findings from other populations, we did not find any unique genetic association between MetS and Arabian populations.

## Introduction

The metabolic syndrome (MetS), which is characterized by insulin resistance, hypertension, obesity, and dyslipidemia (Alberti et al., 2006), is a significant risk factor for atherosclerotic cardiovascular disease (ASCVD) and all-cause mortality (Alberti et al., 2006; Galassi et al., 2006). The prevalence of MetS has been reported as 27.3% in the Arabian Gulf Region (Shin and Jee, 2020). A number of factors can be attributed to this high prevalence, including lifestyle changes and rapid urbanization of the region in the last 40 years (Ramadan, 2015). In one study, Bayoumi and colleagues analyzed data from five large, extended, highly consanguineous Arabian families in Oman to determine the heritability (h2) of MetS and its different components (Bayoumi et al., 2007). The study examined 1,277 individuals in total. They reported that the prevalence of MetS was 23%, and the h2 value was 0.38. The figures are likely to be higher in larger samples due to the highly conserved gene pool in Arabian populations, the high rate of consanguinity in this region as well as the tribal structure of the society and the size of the families in this area (Zayed, 2016).

Several genetic loci are implicated in contributing to the different components of MetS (Barroso and McCarthy, 2019). However, not many genetic studies have focused on MetS as a binary phenotype. For example, searching the GWAS Catalogue (https://www.ebi.ac.uk/gwas/search?query=metabolic%20syndrome) revealed 18 publications on MetS but 193 publications when the search term diabetes was used. Using data from 291,107 individuals from the United Kingdom Biobank, Lind and his colleagues identified 93 independent loci associated with MetS, of which 80 were previously unidentified (Bayoumy et al., 2012). Furthermore, the study demonstrated that MetS is primarily determined by immunity and inflammation pathways and pathways that regulate lipids and lipoproteins. The purpose of this study was to review the current published data on MetS genetics in the Arabian population and suggest future directions.

## Methods

**FIGURE 1:**
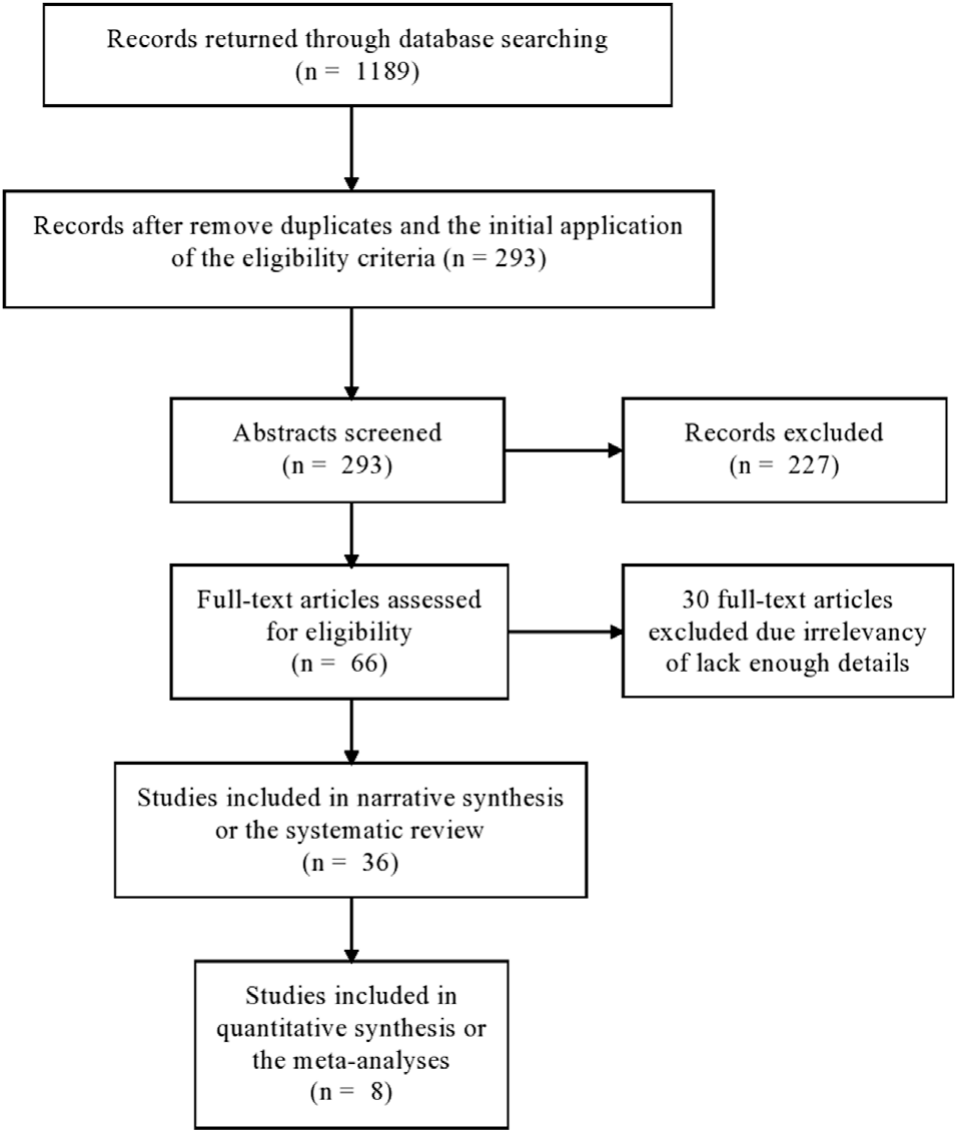
The search strategy of MetS articles in this review.

The flow chart in Figure 1 illustrates the general set-up of the literature search, eligibility check, and extraction of reports, and inclusion of them into the final report.

**Literature search:** In reviewing the published data, these databases were searched: Medline (PubMed or OvidSP), Embase, Scopus, Direct Science, Web of Science, ProQuest, and Google Scholar, from database inception to March 31, 2021, using these search terms and filters:

• “Arab” OR “Arab ancestry” of “Arab ethnicity” OR one of the 22 Arab countries: “Algeria” OR “Bahrain” OR “Comoros” OR “Djibouti” OR “Egypt” OR “United Arab Emirate” OR “Iraq” OR “Jordan” OR “Kuwait” OR “Lebanon” OR “Libya” OR “Mauritania” OR “Morocco” OR “Oman” OR “Palestine” OR “Qatar” OR “Saudi” OR “Saudi Arabia” OR “Somalia” OR “Sudan” OR “Syria” OR “Tunisia” OR “Yemen. ”
• AND “Metabolic syndrome” OR “MetS” OR “Syndrome X” OR “Dysmetabolic syndrome” OR “Insulin resistance syndrome. ”
• AND “Genetic” OR “Genetics” OR “SNP” OR “Single nucleotide polymorphism” OR “Genetic polymorphism” OR “Genetic variant” OR “Genetic variation” OR “Genetic polymorphism” OR “Allele” OR “Genetic locus. ”

A sample search strategy for Medline (via OvidSP) can be found in the Supplementary Figure S1.

**Eligibility:** The eligibility criteria comprised peer-reviewed articles on genetic association with MetS in Arabian populations (candidate-gene studies, genome-wide approaches, or sequencing approaches). In this context, we defined the Arabian population as those who are associated with the Arab League, as shown in the following table. Articles eligible for consideration included case-control studies examining MetS as a dichotomous outcome (MetS versus no MetS), and published in English. The exclusion criteria were studies that did not meet the inclusion criteria, grey literature, duplicate publications, and studies without detailed results, including odds ratios, confidence intervals, and *p* values. We also excluded case report review papers and family association studies. To reduce the risk of selection and methodological bias, studies that reported one of the standard definitions of MetS (IDF, AHA/NHLBI, or NCEP - ATP III) were included in the meta-analysis (Supplementary Table S1). Two authors identified and read an abstract to assess the article’s eligibility and then confirmed by the primary investigator for meeting the criteria for inclusion and exclusion. When a paper did not contain an abstract or provide sufficient information, the full text or supplementary materials were reviewed for suitability. Abstracts were evaluated according to their eligibility as ‘yes,’ ‘no’ or ‘unclear.’ The unclear abstracts were kept until the full-text articles were reviewed before a decision was made regarding inclusion.

**Data extraction:** The full-text data was extracted by three authors (ZA, NA, and MM) after screening the titles and abstracts to determine their eligibility. Any disagreements were resolved by a fourth researcher (WO). Further, detailed data were extracted from standardized data extraction forms, including author affiliations, study designs and settings, study population characteristics, MetS classification criteria, number of cases and controls, obesity determination and measurements, genetic markers, tested alleles, and association analysis methods and outcomes.

**Quality of studies:** To assess the quality of the studies, the Q-Genie (Quality of Genetic Association Studies) tool was applied, which has a scoring system covering eleven different elements, as previously reported (Bayoumy et al., 2012). Studies were classified as poor quality (a score of ≤35), moderate: scores of >35 and ≤45; and good: scores of >45.

**Statistical analysis:** Statistical analysis was performed using R statistical software Version 4.0.3 and Stata software Version 14 (StataCorp, TX, United States). In Stata software, we used the “metan” command. The heterogeneity (H) was determined using the non-central chi (Galassi et al., 2006) (common-effect) distribution for Q and *I* (Galassi et al., 2006) statistics. According to initial analyses, most of the studies had significant among-study heterogeneity (*I* (Galassi et al., 2006) > 50%). Therefore, we conducted the meta-analysis by pooling aggregate data using the random-effects inverse-variance model, which included the DerSimonian-Laird model estimator for tau squared (between-study variance, also known as *τ* (Galassi et al., 2006)) in addition to *I* (Galassi et al., 2006). Furthermore, we generated Forest plots to illustrate the pooled prevalence estimates derived from the meta-analysis calculations. *p* < 0.05 was used as the threshold for statistical significance. The eyeball test (Funnel plot) and Egger’s test were not considered because the number of studies used in the meta-analysis was small ( <10 studies), and the test had a relatively low statistical power to determine whether the observed asymmetry was caused by chance.

## Results

### Literature Search and Study Characteristics

Our search strategy (Figure 1 and Methods section) identified 36 studies that met our inclusion criteria (Bayoumi et al., 2007; Bayoumy et al., 2012; Boumaiza et al., 2012a; Boumaiza et al., 2012b; Ghattas et al., 2013; Mohamed Youssef et al., 2013; Zadjali et al., 2013; Ajjemami et al., 2014; Al-Daghri et al., 2014; Mackawy and Badawi, 2014; Youssef et al., 2014; Ajjemami et al., 2015; Mehanna et al., 2015; Alnory et al., 2016; Mehanna et al., 2016; Elouej et al., 2016a; RaafatAbdRaboh et al., 2016; Elouej et al., 2016b; Elouej et al., 2016c; Khella et al., 2017; Hasan et al., 2017; Lakbakbi El Yaagoubi et al., 2017; Mackawy, 2017; Morjane et al., 2017; Ismail et al., 2019; Ibrahim and Bastawy, 2020; Salami and El Shamieh, 2019; ElGhareeb et al., 2019; Nizam et al., 2019; Boulenouar et al., 2019; Aljaibeji et al., 2020; Osman et al., 2020; Alenad et al., 2020; Hechmi et al., 2020; Kefi, 2021). In most cases, studies were excluded because they lacked detailed results, such as odds ratios, confidence intervals, and *p*-values. The study conducted by Bayoumi et al. did not study genetic markers. However, we included it because it reported the heritability of MetS and its components using data from Oman, which was found to be 0.38 ^5^. Among the 36 association studies, the majority came from Egypt (13 studies) and Tunisia (10 studies) (**Table 1**). The majority of studies adopted the IDF definition of MetS over other definitions like the AHA/NHLBI (Grundy et al., 2005) and the NCEP — ATP III (Alexander et al., 2003) (Supplementary Table S1).

### Genetics of Obesity in Arab Populations

**FIGURE 2:**
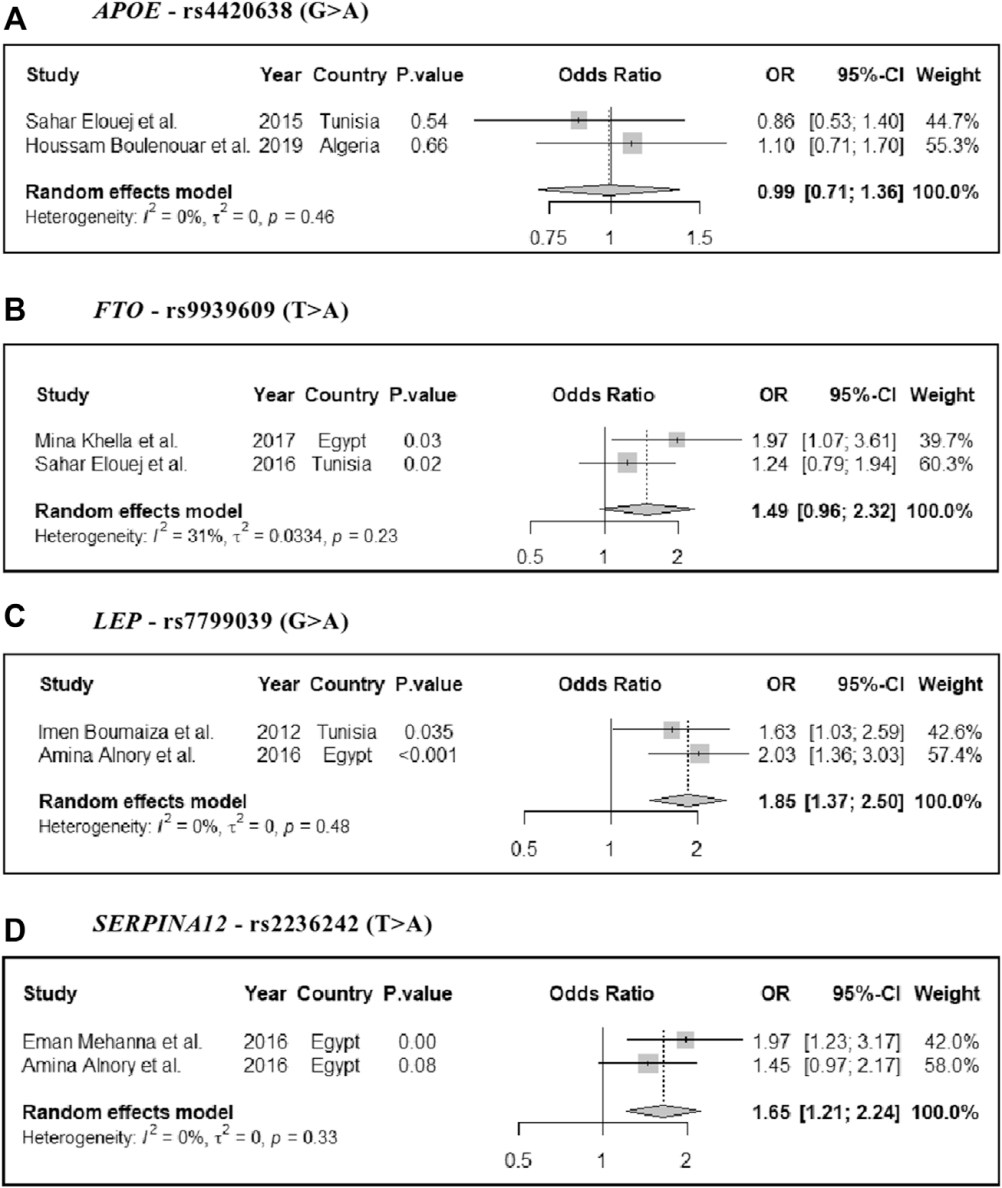
Forest plot of the pooled odds ratios and 95% CI estimates of MetS for four genetic markers: **(A)** *APOE* - rs4420638 (G > A), **(B)** *FTO* - rs9939609 (T > A), **(C)** *LEP* - rs7799039 (G > A), and **(D)** *SERPINA12* - rs2236242 (T > A).

All studies were gene-candidate studies (**Table 1**) that selected genes based on previous literature reports or their predicted functions in relation to MetS development. There was considerable variability in the number of genes selected, ranging from one to six. The most commonly studied genes were *FTO* and *VDR* (both four studies, Supplementary Table S2), whereas the most frequently tested markers were rs10735810 (FokI) and rs1544410 (BsmI); both in the VDR gene (Supplementary Table S3). Four genetic markers found eligible for the meta-analysis studies: rs9939609 (*FTO*), rs4420638 (*APOE*), rs7799039 (*LEP*), and rs2236242 (*SERPINA12*). As all of these indicated a significant heterogeneity [*I* (Galassi et al., 2006)> 50%] (Figure 2), we used the random-effect inverse-variance model for the meta-analysis studies (see the Methods section). Three of these markers indicated increased risk for MetS after calculations of the pooled odds ratios: *FTO*-rs9939609 (OR = 1.49, 95% CI: 0.96–2.32); *LEP*-rs7799039 (OR = 1.85, 95% CI: 1.37–2.5); and *SERPINA12*-rs2236242 (OR = 1.65, 95% CI: 1.21–2.24), Figure 2. These three genes are mainly implicated in obesity or lipid metabolism (Supplementary Tables S4,5).

### Quality of the Studies

In order to assess the quality of the studies, the Q-Genie tool was utilized (Sohani et al., 2016). It is a validated tool of bias and quality for systematic reviews of genetic association studies. The tool employs 11 criteria, covering all association steps, including rationale, sampling, selection of cases and controls, methodology, analysis, and conclusions. The tool indicated that 12 studies had poor quality (a score of ≤35), 13 studies had moderate quality (scores >35 and ≤45); and only eight studies had good quality (scores >45). The detailed quality information for all studies is summarized in Supplementary Table S6. Specifically, three factors affected the study’s quality: the non-technical classification of the genetic variants, the disclosure and discussion of biases, and the size and power calculations. (Supplementary Table S6).

## Discussion

### Main Findings

Genetic studies should be conducted across populations due to differences in linkage disequilibrium (LD)/haplotype blocks and the type and frequency of monomorphic/polymorphic SNPs. Many studies have investigated the genetics of MetS, but few have examined MetS as a binary outcome. In light of the high prevalence of MetS in Arab populations and its components (Shin and Jee, 2020), the current study reviewed the studies that examined MetS in Arabic populations as a binary trait.

In the narrative synthesis, 36 studies were included after several were excluded due to insufficient methodology or statistical analysis information. All of the studies were candidate gene studies, and none of the studies utilized a genome-wide or sequencing approach. Moreover, there was considerable variation in the tested markers and genes, with most studies evaluating a single gene (Elouej et al., 2016b; Khella et al., 2017; Mackawy, 2017) to several genes in others (Elouej et al., 2016c; Osman et al., 2020). The study conducted by Bayoumi et al. reported the heritability of MetS using data from Oman, which was found to be 0.38. Based on their cultural similarity, we expected other Arab populations to have a similar prevalence of MetS.

### Heterogeneity

There were several reasons for heterogeneity between these studies regarding MetS definitions, study populations, sample sizes, research design, and selection of the genetic markers (**Table 1**). For example, while most of the studies focused on diverse populations, a few included only females in their research (Mehanna et al., 2015; Morjane et al., 2017), and one study examined MetS in children (Nizam et al., 2019). Similarly, the proportion of males and females, and the age range, varied in most studies; both of these factors are well-known sources of heterogeneity in meta-analyses (Pei et al., 2016). It is also important to note that the precise definition of the Arab population is challenging since there are different ethnic subgroups within the Arab world. For example, admixture studies revealed that the Qatari population is made up of three major sub-populations derived from Arabic, Persian, and African ancestries (Omberg et al., 2012). It indicates that several factors related to ethnic backgrounds, such as the LD patterns and allele frequencies, have not been considered by most reports. Moreover, heterogeneity in the study was assessed using the heterogeneity statistic *I* (Galassi et al., 2006), which estimates the proportion of the variance in a study due to heterogeneity. Nonetheless, *I* (Galassi et al., 2006) values can be imprecise and possess a substantial bias when the number of studies in a meta-analysis study is small, as in our study (von Hippel, 2015).

### The Reported Genetic Markers

Most of the genetic markers were selected based on their functional predictability related to diabetes, insulin resistance, obesity, or previous reports of their association with metabolic syndrome. The functions and key information for these genes (*FTO*, *APOE*, *LEP*, and *SERPINA12*) are shown in Supplementary Table S4. The STRING database (Szklarczyk et al., 2019) has demonstrated that these genes are involved in several biological processes associated with the development of MetS, including steroid metabolism, lipid metabolism, apoptosis, glucose metabolism, cell signaling, and regulation of inflammatory responses response (Supplementary Table S5). Among the studies included in the meta-analysis study, three markers were found to be significant in the pooled associations with MetS: rs9939609 (odds ratio 1.49); rs7799039 (odds ratio 1.85); and rs2236242 (odds ratio 1.65). Despite several sources of heterogeneity, we observed no significant differences between the pooled and meta-analyzed studies (Figure 2). *FTO* gene encodes alpha-ketoglutarate-dependent dioxygenase, which regulates food intake and energy expenditure (Kalantari et al., 2016). Genetic variants in the *FTO* gene have been associated with many traits and diseases, such as obesity, lipid metabolism, type 2 diabetes, and MetS (Kucher, 2020). *APEO* encodes the glycoprotein apolipoprotein E, which is important in the metabolism of lipoproteins and lipids (Tudorache et al., 2017). In addition, *LEP* encodes leptin, a key hormone responsible for maintaining energy balance and regulating body weight (Pan and Myers, 2018). This indicates that the genes were mainly selected to be studied in association with MetS based upon their roles in obesity or lipid metabolism, not their roles in inflammatory pathways, which have been shown to be key contributors to MetS (Lind, 2019).

However, these associations with MetS are not limited to Arab populations (Teshigawara et al., 2012; Wang et al., 2012; Ghalandari et al., 2015; Albarracín and Torres, 2020; Hardy et al., 2020). For isntance, H Wang and colleagues (Wang et al., 2012) published a meta-analysis of 18 studies regarding the association of genetic variations in the FTO gene with MetS and reported significant pooled associations.

### Quality of the Studies

Considering that the number of studies was small when divided by different genes and genetic variations, we could not determine the degree of publication bias. Consequently, it was challenging to use methods such as GRADE to evaluate the quality of studies. The Q-Genie tool was utilized as a quality assessment tool because genetic studies are unique compared to epidemiological studies, especially regarding bias sources, such as variations related to the collection, handling, and processing of DNA, genotype, and relatedness classification in the population under study (Boumaiza et al., 2012a). Twenty-five studies were rated as having a moderate to poor quality by the Q-Genie tool (67.6%) (Supplementary Table S6). The primary causes of low-quality studies were the sample size, insufficient power calculations, and not taking into account significant sources of bias when designing the study.

Furthermore, several studies have indicated insufficient information about the non-technical classification of genetic variations and how genotyping was performed. Additionally, many studies did not disclose other potential sources of bias (e.g., time-lag bias, attrition bias, recall bias) and how they were handled appropriately.

### Limitations, Strengths, and Recommendations to Improve Future Studies

1) The protocol of this review has not been pre-registered for this (e.g., in PROSPERO), which may introduce potential bias per Cochrane guidelines.
2) There have been no comprehensive studies or genome-wide studies conducted on the association of MetS in any Arab population. Additionally, several studies have been conducted to examine some individual components of MetS rather than MetS as a whole. We recommend establishing a regional consortium that can address this problem and unify the definition of MetS, the study settings, and the analysis plans.
3) There was an underrepresentation of studies from the low- and middle-income regions of the Arab world, especially those that are subject to war and political instability (Iraq, Syria, Libya, Sudan, Yemen, and Palestine).
4) There were several studies with poor design or analysis of the results. In addition, several studies were excluded due to a lack of results detail, such as a lack of odds ratios or confidence intervals. Therefore, we recommend that MetS researchers in the region carefully design their studies, consider potential bias sources, and report results in detail.
5) In this systematic review, a significant concern was using different names for the same marker (e.g., rs1801282 in *PPARγ2* gene or rs2228570 in *VDR* gene), making it difficult to conclude these studies. Therefore, we recommend using common gene or SNP markers nomenclature systems, such as the HUGO Gene Nomenclature for genes and the bSNP Reference SNP (rs or RefSNP) number for genetic markers.
6) Lastly, as reported in Beshyah and colleagues in 2018 (Beshyah et al., 2018), we observed that a significant number of papers are being published in predatory journals. A recent report revealed that the Middle East or Gulf Cooperation Council region is a significant target for predatory journals (Macháček and Srholec, 2021). Researchers are advised to increase their knowledge of predatory journals and become familiar with Beall’s list of these journals and other resources available to them.

We summarized the current literature on the genetic associations of MetS in Arab populations in this review. We found that three markers, FTO-rs9939609, LEP-rs7799039, and SERPINA12-rs2236242, contributed significantly to the pooled risk for MetS development. As a result of considerable variations in study settings and numerous sources of heterogeneity, most of these studies are rated as of low to moderate quality.

## Data Availability

The raw data supporting the conclusions of this article will be made available by the authors, without undue reservation.
