# Supplementary material for: Genetic Studies of Metabolic Syndrome in Arab Populations: A Systematic Review and Meta-Analysis
Source: Front Genet. 2021 Nov 18;12:733746. doi: 10.3389/fgene.2021.733746 (PMC8637276; doi:10.3389/fgene.2021.733746)
Supplement: Supplementary file 1 [file Table5.pdf]

Table S5: STRING results of the combining biological functions and pathways using different combinations of the most frequently studies genes in this study.

**Genes**	**Biological function (pathways)**
APOE|LEP|SERPINA12	Regulation of steroid metabolic process
APOE|LEP|SERPINA12	Regulation of lipid biosynthetic process
APOE|LEP|SERPINA12	Regulation of small molecule metabolic process
APOE|LEP|SERPINA12	negative regulation of biosynthetic process
APOE|LEP|SERPINA12	Regulation of intracellular signal transduction
APOE|LEP|SERPINA12	Extracellular space
APOE|LEP|FTO	Regulation of lipid localization
APOE|LEP|FTO	Regulation of developmental growth
APOE|LEP|FTO	Homeostatic process
APOE|LEP|FTO	Regulation of cell population proliferation
APOE|LEP|FTO	Regulation of cell differentiation
LEP|SERPINA12	Positive regulation of insulin receptor signaling pathway
LEP|SERPINA12	Regulation of gluconeogenesis
LEP|SERPINA12	Positive regulation of phosphatidylinositol 3-kinase signaling
LEP|FTO	Regulation of brown fat cell differentiation
LEP|FTO	Adipose tissue development
LEP|FTO	Regulation of lipid storage
LEP|FTO	Obesity
LEP|FTO	Regulation of system process
APOE|SERPINA12	Regulation of triglyceride metabolic process
APOE|SERPINA12	negative regulation of lipid biosynthetic process
APOE|SERPINA12	Regulation of cholesterol metabolic process
APOE|SERPINA12	Negative regulation of small molecule metabolic process
APOE|LEP	Response to dietary excess
APOE|LEP	Negative regulation of lipid localization
APOE|LEP	Negative regulation of anion transport
APOE|LEP	Regulation of nitric-oxide synthase activity
APOE|LEP	Regulation of cholesterol transport
APOE|LEP	Positive regulation of blood vessel diameter
APOE|LEP	Fatty acid transport
APOE|LEP	Regulation of steroid biosynthetic process
APOE|LEP	Cholesterol metabolic process
APOE|LEP	Regulation of endothelial cell proliferation
APOE|LEP	Cellular lipid catabolic process
APOE|LEP	Positive regulation of ion transport
APOE|LEP	Negative regulation of response to external stimulus
APOE|LEP	Regulation of inflammatory response
APOE|LEP	Blood vessel morphogenesis

APOE|LEP	Small molecule catabolic process
APOE|LEP	Response to toxic substance
APOE|LEP	Regulation of cell activation
APOE|LEP	Endocytosis
