# Supplementary material for: Genetic Studies of Metabolic Syndrome in Arab Populations: A Systematic Review and Meta-Analysis
Source: Front Genet. 2021 Nov 18;12:733746. doi: 10.3389/fgene.2021.733746 (PMC8637276; doi:10.3389/fgene.2021.733746)
Supplement: Supplementary file 2 [file Table4.pdf]

Table S4: Key information about the most frequently studies genes in this study

Gene	Annotation
FTO	Alpha-ketoglutarate-dependent dioxygenase. The product of the gene is dioxygenase that repairs alkylated DNA and RNA by oxidative demethylation. Has highest activity towards single- stranded RNA containing 3-methyluracil, followed by single- stranded DNA containing 3-methylthymine. Has low demethylase activity towards single-stranded DNA containing 1-methyladenine or 3-methylcytosine. Specifically demethylates N(6)-methyladenosine (m6A) RNA, the most prevalent internal modification of messenger RNA (mRNA) in higher eukaryotes.
APOE	Apolipoprotein E. Mediates the binding, internalization, and catabolism of lipoprotein particles. It can serve as a ligand for the LDL (apo B/E) receptor and for the specific apo-E receptor (chylomicron remnant) of hepatic tissues.
SERPINA12	Serpin A12. Is an adipokine that modulates insulin action by specifically inhibiting its target protease KLK7 in white adipose tissues.
LEP	Leptin. Is a key player in the regulation of energy balance and body weight control. Once released into the circulation, has central and peripheral effects by binding LEPR, found in many tissues, which results in the activation of several major signaling pathways. In the hypothalamus, acts as an appetite-regulating factor that induces a decrease in food intake and an increase in energy consumption by inducing anorexinogenic factors and suppressing orexigenic neuropeptides, also regulates bone mass and secretion of hypothalamo-pituitary-adrenal hormones.
