# Supplementary material for: Genetic Studies of Metabolic Syndrome in Arab Populations: A Systematic Review and Meta-Analysis
Source: Front Genet. 2021 Nov 18;12:733746. doi: 10.3389/fgene.2021.733746 (PMC8637276; doi:10.3389/fgene.2021.733746)
Supplement: Supplementary file 3 [file Table1.pdf]

Table S1: Different definition criteria for MetS

Ref	Definition	Criteria
1	**IDF definition**	**<u>Central obesity</u>** (defined as waist circumference* with ethnicity-specific values) **<u>plus any two</u>** of the following four factors:<br>1) Raised triglycerides: ≥ 150 mg/dL (1.7 mmol/L) or specific treatment for this lipid abnormality<br>2) Reduced HDL cholesterol: < 40 mg/dL (1.03 mmol/L) in males< 50 mg/dL (1.29 mmol/L) in females or specific treatment for this lipid abnormality<br>3) Raised blood pressure: systolic BP ≥ 130 or diastolic BP ≥ 85 mm Hg or treatment of previously diagnosed hypertension<br>4) Raised fasting plasma glucose: (FPG) ≥ 100 mg/dL (5.6 mmol/L),or previously diagnosed type 2 diabetes If above 5.6 mmol/L or 100 mg/dL, OGTT is strongly recommended but is not necessary to define presence of the syndrome.
45	**The AHA and NHLBI**	Require **<u>at least three of the following criteria</u>** for the diagnosis of the metabolic syndrome:<br>1) Waist circumference of at least 40 inches (102 cm) in men or 35 inches (89 cm) in women, measured at the top of the iliac crest at the end of a normal expiration<br>2) Triglyceride level of at least 150 mg per dL (1.70 mmol per L), or receiving pharmacologic therapy for elevated triglyceride levels<br>3) HDL cholesterol level of less than 40 mg per dL (1.05 mmol per L) in men or less than 50 mg per dL (1.30 mmol per L) in women, or receiving pharmacologic therapy for reduced HDL cholesterol levels<br>4) Systolic blood pressure of at least 130 mm Hg or diastolic blood pressure of at least 85 mm Hg, or receiving pharmacologic therapy for hypertension<br>5) Fasting glucose level of at least 100 mg per dL (5.6 mmol per L), or receiving pharmacologic therapy for elevated fasting glucose levels.
46	**The NCEP ATP III panel**	**<u>The presence of three or more</u>** of the following risk determinants:<br>1) increased waist circumference (>102 cm [>40 in] for men, >88 cm [>35 in] for women)<br>2) elevated triglycerides (≥150 mg/dl); *3*) low HDL cholesterol (<40 mg/dl in men<br>3) low HDL cholesterol (<40 mg/dl in men, <50 mg/dl in women)<br>4) hypertension (≥130/≥85 mmHg)<br>5) impaired fasting glucose (≥110 mg/dl)
