# Supplementary material for: Genetic Studies of Metabolic Syndrome in Arab Populations: A Systematic Review and Meta-Analysis
Source: Front Genet. 2021 Nov 18;12:733746. doi: 10.3389/fgene.2021.733746 (PMC8637276; doi:10.3389/fgene.2021.733746)
Supplement: Supplementary file 4 [file Table2.pdf]

Table S2: Reported associations between genes and MetS in Arab populations, grouped by genes

Study	Country	Gene
El Yaagoubi et al.,2017	Morocco	*ADCY5*
F. Zadjali et al.,2013	Oman	*ADIPOQ*
Amal Alenad et al.,2020	Saudi Arabia	*ANGPTL8*
Eman Mehanna et al.,2014	Egypt	*APLN*
El Yaagoubi et al.,2017	Morocco	*APO*
Maria Ajjemami et al.,2015	Morocco	*APOA5*
Meriem Hechmi.,2020	Tunisia	
Rym Kefi.,2017	Tunisia	
Maria Ajjemami et al.,2014	Morocco	*APOC3*
Houssam Boulenouar.,2019	Algeria	*APOE*
Sahar Elouej et al.,2016	Tunisia	
Mohamed ElGhareeb et al.,2019	Egypt	*AT1R*
Rasheeba Nizam et al.,2018	Kuwait	*CAV1*
Nervana Bayoumy et al.,2012	Egypt	*CD36*
Sahar Elouej et al.,2016	Tunisia	*CILP2*
El Yaagoubi et al.,2017	Morocco	*DUSP9*
Maivel Ghattas et al.,2013	Egypt	*ERα*
Mina S.Khella et al.,2017	Egypt	*FTO*
Sahar Elouej et al.,2015	Tunisia	
Sahar Elouej et al.,2016	Tunisia	
Sahar Elouej et al.,2015	Tunisia	
Wael Osman et al.,2020	UAE	
El Yaagoubi et al.,2017	Morocco	*G6PC2*
Imane Morjane.,2017	Morocco	*HNF1A*
Sahar Elouej et al.,2016	Tunisia	*INSR*
Sahar Elouej et al.,2016	Tunisia	*KLF14*
Naglaa AbdRaboh et al.,2016	Egypt	*LEP*
Amina Alnory.,2016	Egypt	
Imen Boumaiza et al.,2012	Tunisia	
Imen Boumaiza et al.,2012	Tunisia	*LEPR*
Wael Osman et al.,2020	UAE	*LIPC*
Sahar Elouej et al.,2016	Tunisia	*LRPAP1*
Eman Mehanna et al.,2014	Egypt	*miRNA-146a*
Riad Bayoumi., 2012	Oman	*N/A*
Wael Osman et al.,2020	UAE	*NR1H3*
Hayat Aljaibeji et al.,2020	UAE	*PLCXD3*

SarrajYoussef et al.,2013	Tunisia	*PPARγ2*
Sarraj Youssef et al.,2013	Tunisia	
El Yaagoubi et al.,2017	Morocco	*PROX1*
Amal Machawy et al.,2017	Egypt	*PTPN1*
Eman Mehanna.,2016	Egypt	*RARRES2*
Sherine Ibrahim et al.,2020	Egypt	*RETN*
Imen Boumaiza et al.,2012	Tunisia	
Amina Alnory.,2016	Egypt	*SERPINA12*
Eman Mehanna.,2016	Egypt	
Nagwa Ismail et al.,2018	Egypt	*TCF7L2*
Wael Osman et al.,2020	UAE	*TFAP2B*
El Yaagoubi et al.,2017	Morocco	*UBE2E2*
Amal Mackawy et al.,2014	Egypt	*VDR*
Nasser Al-Daghri et al.,2014	Saudi Arabia	
Hayder Hasan et al.,2016	UAE	
Ali Salami et al.,2019	Lebanon	*VLDLR-KCNV2*
Ali Salami et al.,2019	Lebanon	*ZFPM2*
Sahar Elouej et al.,2016	Tunisia	*ZNF664*
