# Supplementary material for: Genetic Studies of Metabolic Syndrome in Arab Populations: A Systematic Review and Meta-Analysis
Source: Front Genet. 2021 Nov 18;12:733746. doi: 10.3389/fgene.2021.733746 (PMC8637276; doi:10.3389/fgene.2021.733746)
Supplement: Supplementary file 6 [file Table3.pdf]

Table S3: Genetic polymorphisms reported with MetS in Arab populations.

Reference	Gene	Polymorphism	Alleles	Number of studies
29	*ADCY5*	rs11708067	A/G	1
15	*ADIPOQ*	rs17300539	G/A	1
15		rs266729	G/C	1
38	*ANGPTL8*	rs2278426	C/T	1
38		rs737337	T/C	1
22	*APLN*	rs2235306	T/C	1
29	*APO*	rs5128 (C33238C>G)	C/G	1
43	*APOA5*	rs662799	A/G	2
20		rs662799		
44		rs3135506	G/C	1
44		rs651821	T/C	1
20	*APOA6*	c.56C > G	C/G	1
20	*APOA7*	c.553G > T	G/T	1
20	*APOA8*	c.1259 T > C	T/C	1
16	*APOC3*	SstI (3238C> G)	C/G	1
39	*APOE*	APOEε	ε2, ε3. ε4	1
39		rs439401	C/T	1
26		**rs4420638 ***	G/A	2
39		**rs4420638 ***		
34	*AT1R*	A1166C	A/C	1
35	*CAV1*	rs1997623	C/A	1
10	*CD36*	rs1761667	A/G	1
26	*CILP2*	rs10401969	T/C	1
29	*DUSP9*	rs5945326	A/G	1
13	*ERα*	[c.454-397 T/C: PvuII restriction site (rs2234693)]	T/C	1
13		[c.454-351 A/G: XbaI restriction site (rs9340799)]	A/G	1
21	*FTO*	rs1421085	T/C	1
21		rs8057044	A/G	1
25		**rs9939609 ***	T/A	2
27		**rs9939609 ***		
37		rs9939973	A/G	1
29	*G6PC2*	rs560887	A/G	1
42	*HNF1A*	rs1169288	A/C	1
42		rs2464196	G/A	1

42		rs735396	T/C	1
26	*INSR*	rs2059807	C/T	1
26	*KLF14*	rs1562398	G/C	1
24	*LEP*	3’tetranucleotide microsatellite polymorphism	I (short) /II (long)	1
12		**rs7799039 (G-2548A) ***	G/A	2
40		**rs7799039 (G-2548A) ***	G/A	
12	*LEPR*	Q223R	Q/R	1
37	*LIPC*	rs4775041	G/C	1
26	*LRPAP1*	rs762861	G/C	1
23	*miRNA-146a*	rs2910164	G/C	1
37	*NR1H3*	rs10838681	A/G	1
36	*PLCXD3*	rs9292806	G/C	1
36		rs319013	T/G	1
14	*PPARα2*	rs1800206 (Leu162Val)	Leu/Val	1
14		rs1801282 (Pro12Ala)	Pro/Ala	2
19		rs1801282 (Pro12Ala)		
19		Exon 6 C161T	C/T	1
29	*PROX1*	rs340874	A/G	1
30	*PTPN1*	467T>C	T/C	1
41	*RARRES2*	rs17173608	T/G	1
32	*RETN*	+62G>A	G/A	1
11		394C/G	C/G	1
11		420C/G	C/G	1
11		44G/A	G/A	1
11		62G/A	G/A	1
40	*SERPINA12*	**rs2236242 ***	T/A	2
41		**rs2236242***		
31	*TCF7L2*	rs290487	C/T	1
37	*TFAP2B*	rs2206277	T/C	1
29	*UBE2E2*	rs7612463	A/C	1
18	*VDR*	rs10735810 (FokI)	C/T	3
28		rs10735810 (FokI)		
17		rs10735810 (FokI)		
18		rs1544410 (BsmI)	A/G	3
28		rs1544410 (BsmI)		
17		rs1544410 (BsmI)		
28		rs731236 (TaqI)	T/C	2
17		rs731236 (TaqI)		

17		rs7975232 (ApaI)	A/C	1
33	*VEGF*	rs10738760	A/G	1
33	*ZFPM2*	rs6993770	A/T	1
26	*ZNF664*	rs12310367	A/G	1

* Markers in bold are included in the mata-analysis studies
