# Supplementary material for: Genetic Studies of Metabolic Syndrome in Arab Populations: A Systematic Review and Meta-Analysis
Source: Front Genet. 2021 Nov 18;12:733746. doi: 10.3389/fgene.2021.733746 (PMC8637276; doi:10.3389/fgene.2021.733746)
Supplement: Supplementary file 7 [file Image1.pdf]

The search strategy for the Medline database (via OvidSP)

Database: Medline (Ovid MEDLINE® Epub Ahead of Print, In-Process & Other Non-Indexed Citations, Ovid MEDLINE® Daily and Ovid MEDLINE®) from database inception to March 2021. [Metabolic syndrome/ MetS/ Syndrome X / Dysmetabolic syndrome /Menstruation /Menarche / Insulin resistance syndrome] AND [Genetic / Genetics/ Genetic association/ Genetic Factors /Molecular Factors / SNP Single nucleotide polymorphism / Genetic polymorphism/ Genetic variant/ Genetic variation/ Genetic polymorphism/ Allele/ Genetic locus/ Genetic Association] AND [Jordan or Iraq or Syria or "Saudi" or "Saudi Arabia" or Kuwait or Yemen or Qatar or Bahrain or Oman or Lebanon or Sudan or Tunisia or Algeria or Libya or Morocco or Somalia or Egypt or Emirates or United Arab Emirates or Comoros or Djibouti or Mauritania OR Jordanian or Iraqi or Syrian or Kuwaiti or Yemeni or Qatari or Bahraini or Omani or Lebanese or Sudanese or Tunisian or Algerian or Libyan or Moroccan or Somali or Somalian or Egyptian or Emirati or Arab OR Middle East OR Arab countries]
